# Anti-malarial activity of traditional Kampo medicine *Coptis* rhizome extract and its major active compounds

**DOI:** 10.1186/s12936-020-03273-x

**Published:** 2020-06-08

**Authors:** Awet Alem Teklemichael, Shusaku Mizukami, Kazufumi Toume, Farhana Mosaddeque, Mohamed Gomaa Kamel, Osamu Kaneko, Katsuko Komatsu, Juntra Karbwang, Nguyen Tien Huy, Kenji Hirayama

**Affiliations:** 1 Department of Immunogenetics, Institute of Tropical Medicine (NEKKEN), Nagasaki University, 1-12-4 Sakamoto, Nagasaki, 852-8523 Japan; 2 Program for Nurturing Global Leaders in Tropical and Emerging Infectious Diseases, Graduate School of Biomedical Sciences, Nagasaki University, 1-12-4 Sakamoto, Nagasaki, 852-8523 Japan; 3 School of Tropical Medicine and Global Health, Nagasaki University, 1-12-4 Sakamoto, Nagasaki, 852-8523 Japan; 4 Department of Clinical Product Development, Institute of Tropical Medicine (NEKKEN), Nagasaki University, 1-12-4 Sakamoto, Nagasaki, 852-8523 Japan; 5 Section of Pharmacognosy, Institute of Natural Medicine, University of Toyama, Toyama, Japan; 6 Faculty of Medicine, Minia University, Minia, 61519 Egypt; 7 Department of Protozoology, Institute of Tropical Medicine (NEKKEN), Nagasaki University, 1-12-4 Sakamoto, Nagasaki, 852-8523 Japan; 8 Institute of Research and Development, Duy Tan University, Da Nang, 550000 Vietnam

**Keywords:** Herbal medicine, Kampo, Antimalarial

## Abstract

**Background:**

Herbal medicine has been a rich source of new drugs exemplified by quinine and artemisinin. In this study, a variety of Japanese traditional herbal medicine (‘*Kampo*’) were examined for their potential anti-malarial activities.

**Methods:**

A comprehensive screening methods were designed to identify novel anti-malarial drugs from a library of Kampo herbal extracts (n = 120) and related compounds (n = 96). The anti-malarial activity was initially evaluated in vitro against chloroquine/mefloquine-sensitive (3D7) and-resistant (Dd2) strains of *Plasmodium falciparum*. The cytotoxicity was also evaluated using primary adult mouse brain cells. After being selected through the first in vitro assay, positive extracts and compounds were examined for possible in vivo anti-malarial activity.

**Results:**

Out of 120 herbal extracts, *Coptis* rhizome showed the highest anti-malarial activity (IC_50_ 1.9 µg/mL of 3D7 and 4.85 µg/mL of Dd2) with a high selectivity index (SI) > 263 (3D7) and > 103 (Dd2). Three major chlorinated compounds (coptisine, berberine, and palmatine) related to *Coptis* rhizome also showed anti-malarial activities with IC_50_ 1.1, 2.6, and 6.0 µM (against 3D7) and 3.1, 6.3, and 11.8 µM (against Dd2), respectively. Among them, coptisine chloride exhibited the highest anti-malarial activity (IC_50_ 1.1 µM against 3D7 and 3.1 µM against Dd2) with SI of 37.8 and 13.2, respectively. Finally, the herbal extract of *Coptis* rhizome and its major active compound coptisine chloride exhibited significant anti-malarial activity in mice infected with *Plasmodium yoelii* 17X strain with respect to its activity on parasite suppression consistently from day 3 to day 7 post-challenge. The effect ranged from 50.38 to 72.13% (P < 0.05) for *Coptis* rhizom*e* and from 81 to 89% (P < 0.01) for coptisine chloride.

**Conclusion:**

*Coptis* rhizome and its major active compound coptisine chloride showed promising anti-malarial activity against chloroquine-sensitive (3D7) and -resistant (Dd2) strains in vitro as well as in vivo mouse malaria model. Thus, Kampo herbal medicine is a potential natural resource for novel anti-malarial agents.

## Background

Malaria is still considered as a critical health problem in some areas of the world including tropical and subtropical parts. In 2018, 228 million cases of malaria resulted in 405,000 death, of which 93% of the cases and 94% of deaths were in the World Health Organization (WHO) African region [1]. Although lots of efforts have been done, no effective vaccine is available to combat malaria, therefore, chemotherapy and vector control is still the main strategy to counter the parasite [2–5]. Successful malaria control can be achieved through the treatment with efficient anti-malarial drugs, such as quinine and chloroquine (CQ) [6, 7]. However, the inappropriate use of CQ led to the emergence and spread of CQ-resistant *Plasmodium falciparum* parasites which resulted in reducing CQ’s usage for the prophylaxis and treatment for malaria in the late 1970s [8–10]. As a result, artemisinin-based combination therapy (ACT) is highly recommended as a first-line therapy instead of CQ in treating uncomplicated falciparum malaria. However, *P. falciparum* has been recently reported to be resistant to artemisinin in Greater Mekong Sub-region [11–14].

Traditional medicine has been known for centuries and has been used to treat the myriads of ailment [15]. Numerous traditional medicines were derived from the plant-based herbal medicine, namely aspirin from willow bark [16], digoxin from foxglove [17], and morphine from the opium poppy [18]. Interestingly, it persists as a crucial source of drug discovery [15]. Furthermore, the use of herbal medicine for isolation of the natural product from herbal medicine has received increasing attention. It also represented a potential source of the conventional anti-malarial drug [19, 20], such as quinine which was isolated from *Cinchona* bark [21–23] and the use of *Artemisia annua* for isolation of artemisinin [24]. In Africa, herbal medicines are one of the most common traditional medicine and nearly 80% has been utilized as primary health care. Thus, safe and effective herbal medicine should be provided to expand the access to health care service as one-third of the population lack access to essential medicine [25]. Recently, the use of herbal medicine attracts the scientist due to the minimal side effect, lack of modern curative therapy for several chronic diseases, the emergence of microbial resistance, and the needed huge investment for modern drug development. On top of that, the pharmaceutical industries have changed their attention into using herbs as a source of ideal candidates and resurgence their approach in favor of current drug development [26].

Kampo is a Japanese traditional therapeutic system that originated from Chinese traditional medicine [27, 28]. In Japan, Kampo together with modern medicine are usually used in chronic diseases mainly [28]. Not only herbal medicine but also massage, moxibustion, acupuncture and acupressure are included [29, 30]. Each formula among 148 Kampo formulas covered by the Japanese Health Insurance systems has a specific clinical indication for a specific disease and/or symptoms [31]. Moreover, Kampo medicine has been prescribed by over 80% of the physician in Japan and integrated into modern medicine due to high safety and quality [32].

It has been also well-tolerated to human use for thousands of years [33]. Therefore, in this study, 120 Kampo herbal extracts and 96 related compounds were screened for their anti-malarial activity in vitro by using chloroquine/mefloquine-sensitive (3D7) and -resistant (Dd2) strains of *P. falciparum*. After confirmation of *Coptis* rhizome extract, its major compound coptisine chloride, and related formulas having strong activity in vitro, those were further evaluated for their in vivo anti-malarial activity using *Plasmodium yoelii* strain 17X mouse malaria model. To check the presence of *Coptis* rhizome derived compounds in plasma, blood was collected from mice after oral administration of *Coptis* rhizome and Orengedokuto, and analysed by liquid chromatography–mass spectrometry (LC–MS).

## Methods

### In vitro culture of *Plasmodium falciparum*

*Plasmodium falciparum* CQ/mefloquine (MQ)-sensitive (3D7) and -resistant (strain Dd2) strains were originally obtained from Dr. Louis Miller, NIH, USA. The parasites were maintained with 2% haematocrit type O^+^ red blood cells (RBCs) in RPMI-1640-based complete medium (CM) supplemented with 5% AB^+^ human serum (prepared from plasma), 0.25% AlbuMax I (Gibco, Waltham, MA), 12.5 µg/mL gentamycin, and 200 mM hypoxanthine at 37 °C under mixed gas (5% CO_2_, 5% O_2_, and 90% N_2_) condition basically as described [34]. Japanese Red Cross Society was responsible for supplying RBCs and human plasma (number: 28J0060).

### Isolation and culture of primary adult mouse brain cells

Primary adult mouse brain (AMB) cells were isolated and established in NEKKEN Bio-Resource Center, Institute of Tropical Medicine, Nagasaki University as described [35]. The primary cells, which were passaged several times to be adapted to in vitro condition, were maintained in RPMI-1640 media supplemented with 10% fetal bovine serum, penicillin/streptomycin solution (100 units/mL penicillin G, 100 mg/mL streptomycin sulfate) (Wako Pure Chemicals Industrial Ltd, Osaka, Japan) and incubated at 37 °C under 5% CO_2_. The primary cells for cytotoxicity assay were used after three passages.

### Kampo crude drug extracts, compounds, and formula (extracted from *Coptis japonica*)

A Kampo library containing 120 herbal extracts (10 mg/mL), 96 Kampo-related active compounds including three compounds (coptisine chloride, berberine chloride, and palmatine chloride), and powder of experimental Orengedokuto (a Kampo formula containing an aqueous extraction of four medicinal plants, including *Coptis* rhizome*, Phellodendron* bark*, Scutellaria* root, and *Gardenia* fruit, which were blended in the ratio of 3:2:2:2, respectively) were provided by the Institute of Natural Medicine (WAKANKEN), at the University of Toyama as described [36] and stored at − 80 °C. All the herbal extracts were dissolved in ultra-pure water (UPW) generated by Milli-Q (Merck KGaA, Darmstadt, Germany). Compounds were preserved at a concentration of 2 mM dissolved in dimethyl sulfoxide (DMSO; Wako Pure Chemicals Industrial Ltd) solution, the most common solvent for chemicals. For in vivo assays, powder of Orengedokuto and *Coptis* rhizome, as well as chloroquine, were dissolved in distilled water (DW) for oral administration.

### In vitro anti-malarial assay (first screening)

It was done by seeding the *P. falciparum* cultures (0.75% parasitaemia and 2% haematocrit) on 96-well clear flat-bottom plates (Thermo Fisher Scientific, Rochester, NY) and exposed it to Kampo herbal extracts (final concentration of 500 µg/mL). The final UPW solution was less than or equal to 5% of the culture volume, which had no inhibitory effect on parasite growth. CQ (Sigma-Aldrich, St. Louise, MO) and artesunate (Shin Poong Pharm Co, Seoul, South Korea) were used as positive controls (10 µM–0.508 nM), while 5% UPW was used as negative control. The culture plates were kept at 37 °C under mixed gas (90% nitrogen, 5% oxygen and 5% CO_2_) condition for 48 h. Each in vitro experiment was performed in duplicated wells and repeated twice. The inhibition was obtained by dividing the parasitaemia of test samples by the average of the negative controls.

### In vitro dose response assay

The dose–response assay was performed for samples that showed more than 50% inhibition in the first screening to obtain the 50% inhibitory concentration (IC_50_; 10^(log(A/B) × (50‒C)/(D‒C) + log(B)^, where A represented the lowest concentration value at which the percentage inhibition showed greater than 50%, B was the highest concentration value at which the percentage inhibition showed less than 50%, C was the percentage inhibition value of the sample at concentration B, and D was the percentage inhibition value of the sample at concentration A). For the herbal extracts/Kampo formula, and for the compounds in the library, the anti-malarial activity was analysed using a serial dilution of test samples at 500 µg/mL–25.4 ng/mL or at 20 µM–0.619 nM respectively. Artesunate (10 µM–0.508 nM) for 3D7 and CQ (10 µM–0.508 nM) for Dd2 were served as positive controls, while UPW (final 5%) or DMSO (final 0.5%) were assigned as negative controls. The final concentration of DMSO for all tested compounds, negative and positive controls were adjusted to 0.5%.

A SYBR Green based microfluorometric method was used to quantify parasite level as previously described [37]. Briefly, after 48 h of incubation with herbal extracts or compounds, we added 100 µL of lysis buffer to RBCs by using 20 mM Tris, 10 mM EDTA, 0.01% saponin (wt/vol), and then we added 0.1% Triton X-100 (vol/vol), in pH 7.5 as well as 1 × the final concentration of SYBR Green—I (Lonza, Rockland, ME) into each well. The plates were then incubated at room temperature for 1 h with gentle agitation. Finally, the relative fluorescence unit (RFU) per well was detected using a multilabel plate reader (ARVO 1430; Perkin Elmer, Waltham, MA) with 485–515 nm for 0.1 s per exposure.

### Cytotoxicity assay

Cytotoxicity was initially screened at 500 µg/mL for herbal extracts and 20 µM for compounds. AMB cells (1 × 10^4^ cell) were seeded in a 96-well plate (black plate with clear bottom) and incubated at 37 °C in a CO_2_ incubator for 48 h. Then, herbal extracts, compounds, or their negative controls were added, and the cells were further incubated for 48 h. To examine the cell viability (%), 10 µL of Alamar Blue solution (10%, Funakoshi Co., Tokyo, Japan) were added into each well and the cells were incubated for 2 h. Then the fluorescence intensity of each well was measured using a multilabel plate reader at 590 nm for 0.1 s per exposure. The concentration of drug required to reduce cell viability by 50% (CC_50_; 10^(log(A/B) × (50‒C)/(D‒C) + log(B)^, where A represented the lowest concentration value at which the percentage viable cell showed greater than 50%, B was the highest concentration value at which the percentage viable cell showed less than 50%, C was the percentage viable cell value of the sample at concentration B, and D was the percentage viable cell value of the sample at concentration A) was determined for samples that showed less than 50% viability in the initial screening. The assays of duplicated well were performed twice independently. IC_50_ and CC_50_ values were used as an indicator of in vitro anti-malarial activity and an indicator of cytotoxicity in AMB cells. The curve was plotted using GraphPad Prism 6 (GraphPad Software, Inc., San Diego, CA). Selectivity index (SI) was calculated by dividing CC_50_ value by IC_50_ value.

### Assessment of anti-malarial activity in mouse model

A Kampo herbal extract (*Coptis* rhizome) and formula (Orengedokuto) exhibited in vitro anti-malarial activity were tested for in vivo activity against *P. yoelii* strain 17X in a mouse model. Female of 6–7 weeks C57BL/6 N mice (SLC Japan), weighing 18–20 g, were used. The mice were kept in a clean room under conventional conditions then were acclimatized for 1 week before the experiments.

The *P.* *yoelii* 17X strain was provided by Dr. Tetsuo Yanagi, of National Bio-Resource Center (NBRC), NEKKEN, Nagasaki University, Nagasaki, Japan. and maintained by successive serial passage in mice of study. The parasite was maintained frozen at − 80 °C. For each individual assay, an aliquot was injected intraperitoneally (IP) in a mice, and infected donor mice were produced after three in vivo passage. A female C57BL/6 N mouse previously infected with *P. yoelii* and having parasitaemia levels of 20 to 30% were used as a parasite donor. At day 0, mice were injected IP with 0.2 mL of infected blood suspension containing 1 × 10^4^*P. yoelii* parasitized red blood cells obtained from the tail vein of *P. yoelii* infected donor mouse. The *P. yoelii* infected blood was diluted in physiological saline and injected via syringes.

To evaluate the anti-malarial effect of both *Coptis* rhizome and Orengedokuto, infected mice were randomly distributed into four groups of five individuals per cage. Tested drug and CQ were dissolved in DW. Each groups received the drugs 2 h after infection with *P. yoelii* on day 0 and continued daily for 7 days. Animals in test groups were treated twice a day with 365 mg/kg/day of Orengedokuto (Kampo formula) and 122 mg/kg/day of *Coptis* rhizome (Herbal extract) in 0.2 mL solution by oral administration. CQ groups, served as a positive control, received a dose of 10 mg/kg/day and DW groups as a negative control, received 0.2 mL. Amount of dosage is calculated according to the normal usage for humans. Moreover, blood was collected after 1 h from mice treated with *Coptis* rhizome and Orengedokuto to detect berberine, palmatine, and coptisine.

For the in vivo anti-malarial evaluation of the coptisine chloride (Toronto Research Chemicals (TRC), North York, Canada), coptisine chloride, and CQ were dissolved in DW. Three groups of mice were injected intraperitoneally with 0.2 ml of the test sample (30 mg/kg/day Coptisine chloride), positive control (10 mg/kg/day CQ), and negative control (DW) 2 h after infection with *P. yoelii*. The same dose of injection was performed once a day until day 6. The IP route of administration was used since the previous study revealed that coptisine has low oral bioavailability and poorly absorbed through gastrointestinal tracts [38, 39].

On day 3 (72 h post-infection), the parasitaemia level were determined by Giemsa-staining of the tail vein blood smears that was characterized by random counting of the number of parasitized erythrocytes on randomly selected fields of the slide under microscopy of 2000–4000 erythrocytes when parasitaemia was low (≤ 10%) or up to 1000 erythrocytes when parasitaemia was higher.

Results of the in vivo anti-malarial activity were expressed as a mean ± standard deviation (SD) and the comparison of parasitaemia was determined by using a Student’s *t-*test in Microsoft Excel 2016 (Microsoft, USA). The statistical significance level was set at *P *< 0*.05* for all tests. The different field on each slide was examined to calculate the average parasitaemia as shown below.

$$Percentage \, parasitaemia = \, \left( {{\text{Number of parasitized RBC}}/{\text{ Total number of parasites}}} \right) \, \times 100$$

The average percentage of parasite growth suppression was calculated by comparing percentage parasitaemia suppression of the test group with respect to the control according to the equation:

$$Percentage \, suppression = \, \left( {{\text{Mean parasitaemia of negative control }}{-}{\text{ Mean parasitaemia of treated group}}} \right)/ \, \left( {\text{Mean parasitaemia of negative control group}} \right) \, \times 100$$

### Preparation of plasma samples

Healthy 6 weeks old female mice that were subjected to overnight fasting were used for this study. To analyse berberine, coptisine, and palmatine after oral administration of *Coptis* rhizome and Orengedokuto, doses of 122 mg/kg and 365 mg/kg were used for each group, respectively. Five mice per cage were used for each tested drugs. One hour after administration, the blood samples were collected from the tail vein with heparin and centrifuged at 1000×*g* for 20 min to yield plasma sample. Plasma samples were stored at − 80 °C. Before analysis, thawed plasma samples were mixed with methanol with 0.05% (vol/vol) formic acid for 15 min and centrifuged at 14,000 rpm for 15 min. The supernatant was transferred into an Amicon Ultra filter (molecular weight cut-off of 10 kDa, Millipore Corporation), and centrifuged at 14,000 rpm for 60 min at 4 °C. The filtrate was evaporated and redissolved with 50 µL of 30% (vol/vol) MeOH in water to prepare LC–MS sample. LC–MS analyses were conducted with ODS Atlantis T3 (3 μm, 2.1 × 150 mm) column and Shimadzu LCMS system (Shimadzu, Tokyo, Japan) consisting DGU-20A5 on-line degasser, LC-20AD pumps (2 units), SIL-20A autosampler, CTO-20A column oven, SPD-M20A PDA detector, and hybrid ion trap time-of-flight (IT-TOF) mass spectrometer equipped with an ESI (electrospray ionization) interface and chromatogram data were acquired and processed by LCMS Solution (ver. 3.81, Shimadzu). Gradient elution of two solvent mixture consisting of 0.1% (vol/vol) formic acid in water (mobile phase A) and 0.1% (vol/vol) formic acid in acetonitrile (mobile phase B) was run at a flow rate of 0.2 mL/min under the following gradient program: 10% B (0–2 min), 10–100% B (2–20 min), 100% B (20–25 min), 100–10% B (25–26 min), and 10% B (26–36 min). TOF Analyzer was calibrated by sodium trifluoroacetate solution. Data was acquired using the following parameters: detector voltage, 1.80 kV; probe voltage, + 4.5 kV (positive mode) or − 3.5 kV (negative mode); nebulizing gas flow, 1.5 L/min.; drying gas pressure, 100 kPa; temperature for CDL (curved desolvation line) and heat block, 200 °C; ion accumulation time, 30 ms.; scanning range, m/z 100–2000. The temperature of the column oven was set at 40 °C and the injection volume was 5 μL.

### Ethics statement

Human RBCs and plasma were obtained and used after the approval (Number: 15 12 03 146-2) by the institutional ethical review board of Institute of Tropical Medicine, Nagasaki University. The animals in this study were handled according to the international guidelines and institutional guideline of Nagasaki University for the use and maintenance of experimental animals and used after approval (number 1710061412) by the institutional ethical review board of Institute of Tropical Medicine, Nagasaki University.

## Results

### Initial in vitro screening of anti-malarial activity and cytotoxicity of 120 Kampo herbal extracts, related compounds, and Kampo formula

Initially the in vitro anti-malarial activity of Kampo herbal extracts and their related compounds (Additional file 1: Table S1, Table S2) were tested against CQ/MQ-sensitive (3D7) strain of *P. falciparum.* Of 120 herbal extracts, *Coptis* rhizome demonstrated good anti-malarial activity against *P. falciparum* 3D7 (IC_50_ = 1.9 µg/mL) with the minimal toxicity (CC_50_ > 500 µg/mL, SI > 263) (Additional file 1: Table S3). Due to the lowest IC_50_ and high SI, *Coptis* rhizome was further evaluated against *P. falciparum* Dd2 strain and IC_50_ and SI were determined to be 4.85 µg/mL and > 103, respectively (Table 1). Furthermore, the Kampo formula Orengedokuto that contains a high amount of *Coptis* rhizome by percentage weight was selected and evaluated against CQ/MQ-sensitive (3D7) and resistant (Dd2) strain of *P. falciparum.* This formula was received from Institute of natural medicine (WAKANKEN) at the University of Toyama and Tsumura Company (Additional file 1: Table S4) as the content of active ingredient is different. As a result, the IC_50_ of the former was identified to be 3.1 and 6.34 µg/mL against 3D7 and Dd2, respectively. While sample from Tsumura Company showed 36 and 104 µg/mL against 3D7 and Dd2, respectively.

**Table 1:** *In vitro* anti-malarial activities and the cytotoxicities of *Coptis* rhizome and three chlorinated compounds representing its major bioactive components

Name		IC_50_		CC_50_	SI	
		3D7	Dd2		3D7	Dd2
Crud drug extract (µg/mL)						
*Coptis rhizome*		1.9 ± 0.84	4.85 ± 2.33	> 500	> 263	> 103
Compounds (µM)						
Coptisine chloride		1.1 ± 0.05	3.1 ± 0.07	41.1	37.8	13.2
Berberine chloride		2.6 ± 1.22	6.3 ± 0.47	8.6	3.3	1.3
Palmatine chloride		6.0 ± 3.4	11.8 ± 1.62	> 100	> 16.7	> 8.5

Values are the mean from two independent experiments performed in duplicate

IC_50_, 50% inhibitory concentration

CC_50,_ 50% cytotoxic concentration using adult mouse brain cells

SI, selectivity index

### In vitro anti-malarial activity and cytotoxicity of three major bioactive components of *Coptis* rhizome

Because of its highest anti-malarial activity and SI, *Coptis* rhizome, as well as its related compounds, are shown in the supplemental table (coptisine, berberine, and palmatine) these three compounds of the test samples were further investigated against 3D7 and Dd2 strains of *P. falciparum.* IC_50_ values for these components were 1.1, 2.6, and 6.0 µM against 3D7 strain and 3.1, 6.3, and 11.8 µM against Dd2, respectively (Table 1). CC_50_ values were 41.1 µM, 8.64 µM, and > 100 µM, respectively. Thus, the SI of these components were 37.8, > 3.3, and > 16.7 against 3D7 strain and 13.2, 1.3, and 8.5 against Dd2 strain, respectively (Table 1).

### In vivo anti-malarial activity

**Fig. 1:**
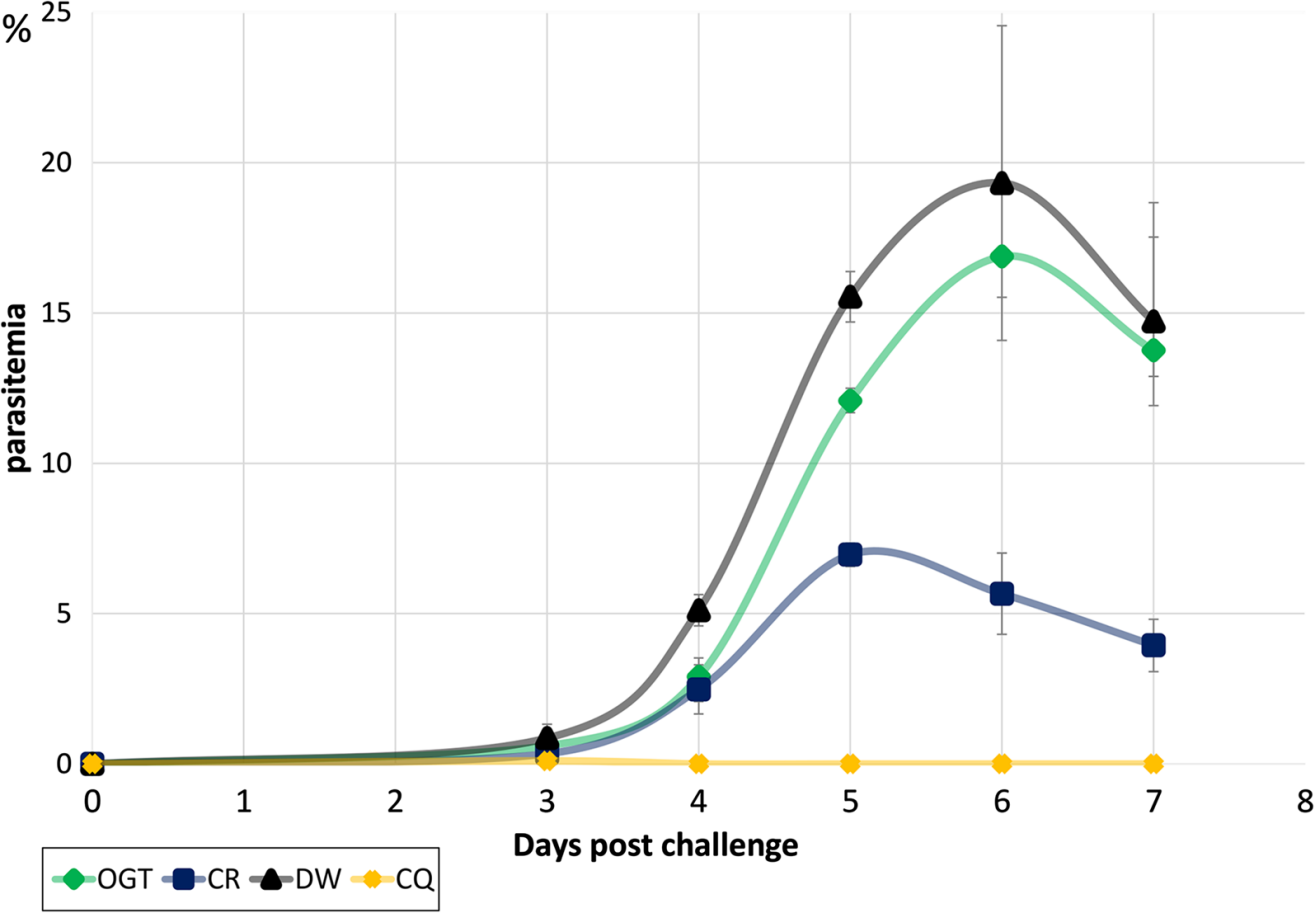
Kinetics of parasitaemia with or without administration of test samples. The above figure indicates the average group parasitaemia of *Coptis* rhizome (CR) and Orengedokuto (OGT) compared with negative control (DW) and positive control (CQ). On day 0, all mice were injected 1 × 10^4^*Plasmodium yoelii* 17X strain intraperitoneally. Two hrs post-challenge, two tested drugs, negative and positive control were administered orally. On day 3 (72 post-challenge) parasitaemia was determined. The x-axis is days after parasite infection while the y-axis shows the percentage of iRBCs

Results of the in vivo malarial suppression test of *Coptis* rhizome and Orengedokuto in mice infected *P. yoelii* 17X strain are summarized in the supplementary file (Additional file 1: Table S5). The parasite density of *Coptis* rhizome revealed low as compared with the negative control (DW) and its parasite suppression were observed from 50.38 at day 3 to 72.13% at day 7 post-challenge (Additional file 1: Table S5). *Coptis* rhizome-treated mice showed significant anti-malarial activity consistently throughout the entire test period to that of negative control (*P *< 0*.05* on day 4 and *P *< *.0* *l* on day 3, 5, 6 and 7) (Fig. 1, Additional file 1: Table S5). Mice treated with CQ significantly suppress the parasitaemia and showed the most potent anti-malarial activity (0% parasitaemia and 100% suppression).

In addition, to analyse the presence of major components derived from *Coptis* rhizome, mice plasma treated with *Coptis* rhizome and Orengedokuto were analysed by LC–MS. Five mice were used in each treatment group. After oral administration of *Coptis* rhizome, the signal of berberine was observed in the plasma from four mice at *m/z* 336.1 and retention time (*t*_R_) 14.0 min. The signals of palmatine (*m/z* 352.1, *t*_R_ 13.8 min) and coptisine (*m/z* 320.1, *t*_R_ 13.0 min) were observed in three plasma samples. In the same way, after oral administration of Orengedokuto, the signal of berberine, palmatine, and coptisine were detected in five, three, and one mice, respectively. Comparing the signal intensities of these three components, the signal of berberine was relatively stronger than the other two compounds (Additional file 1: Table S6, Figure S1).

**Fig. 2:**
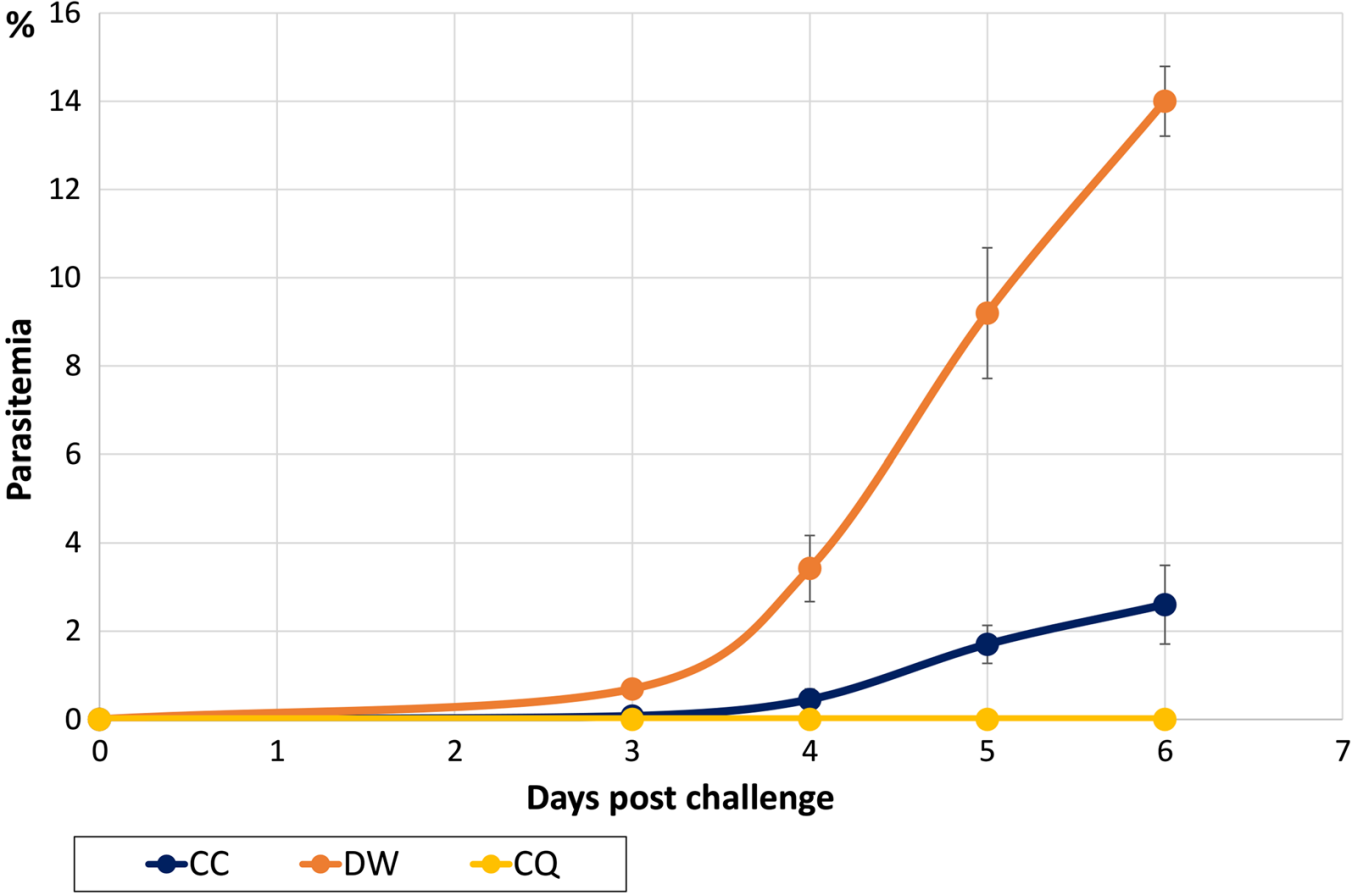
Kinetic of parasitaemia with or without administration of tested samples. The above figure indicates the average group parasitaemia of coptisine chloride (CC) compared with negative control (DW) and positive control (CQ). On day 0, all mice were injected 1 × 10^4^*P. yoelii* 17X strain intraperitoneally. Two hrs post-challenge, tested drug, negative and positive control were administered via intraperitoneally. On day 3 (72 h post-challenge) parasitaemia was determined. The x-axis is days after parasite infection while the y-axis shows the percentage of iRBCs

Figure 2 showed that coptisine chloride suppressed the parasitaemia, which displayed a mean percentage suppression throughout the entire test period (*P * <  *0.1* on days 3 and 4, and *P *< 0*.001*, on days 5 and 6) (Fig. 2, Additional file 1: Table S7). The parasitaemia suppression (%) of mice treated with coptisine chloride was 89%, 87%, 82%, and 81% at days 3, 4, 5, and 6, respectively.

## Discussion

Since *P. falciparum* has quickly acquired resistance against currently available all anti-malarials [40–42], it is urgently required to develop novel anti-malarial drugs. Here it is found that *Coptis* rhizome showed 1.9 µg/ml and 4.9 µg/ml of IC_50_ and > 263 and > 103 SI for Chloroquine sensitive and resistant *P. falciparum* strains, respectively. Furthermore, three chemical compounds (coptisine, berberine, and palmatine), which are related to *Coptis* rhizome exhibited anti-malarial activity with IC_50_ less than 12 µM. These compounds belong to the berberine alkaloidal family and share the same isoquinoline skeletons, which is similar to quinoline skeleton found in anti-malarial drug quinine. This structural similarity to quinine is an important indicator for their anti-malarial activity.

*Coptis* rhizome is one of the components of a formula, Orengedokuto, which has been used to treat inflammatory disease [43], and berberine is strongly suggested to be responsible for its anti-inflammatory effect [44, 45]. In this study, it has confirmed the in vivo anti-malarial activity of *Coptis* rhizome, but the Orengedokuto, whose content [36] is 33.3% *Coptis* rhizome did not show a comparable effect on the reduction of parasitaemia. Because of those mice who got Orengedokuto showed damage in general condition, this formula might contain some interfering components on anti-malarial efficacy. Although this formula is available over the counter and is not necessary to check its safety issue, it is difficult to bring it directly to human trials.

This in vitro finding of *Coptis* rhizome and its bioactive compounds strongly supports a previous report [46]. However, their anti-malarial activity showed much lower IC_50_ using exactly a similar lot of extract and compounds provided from the same KAMPO library of Toyama University. One of the differences between the two institutions is an incubation time of co-culture in vitro before the estimation of parasite number. The previous report used 72 h, but the present study used 48 h. The IC_50_ difference between the 48 h and 72 h may result from time of action. The former method detects the merozoite invasion and subsequent parasite growth from 32 to 46 h and, the latter one detects the mature trophozoites and schizonts, respectively [47]. In addition, the cytotoxicity of berberine showed much lower CC_50_ (CC_50_ 8.3 µM) than the previous one [46]. Other study also reported high levels of cytotoxicity of berberine using murine macrophage (CC_50_ 27.3 µg/mL) [48], or MCF-7 cell (CC_50_ 36.0.91 µg/mL) [49], and 8.75 µg/mL [50].

The in vivo results of coptisine chloride remarkably suppress the parasitaemia of greater than 80%, and the density of parasitaemia was significantly lower than the negative control (*P *< 0*.01*). As previously reported, coptisine had wide verities of activities such as inducing apoptosis in human colon cancer [51], inhibiting inflammatory response of mast cell [52], and antidiabetic [53]. However, this is the first report of the coptisine chloride to have an in vivo anti-malarial activity. After the oral administration of *Coptis* rhizome and Orengedokuto, coptisine were detected in 1/5 and 3/5 of mice, respectively, and the signal of coptisine in plasma samples were relatively lower than that of berberine. The content of coptisine is approximately 1/16 of coptisine in *Coptis* rhizome [54]. Therefore, this results reflect the contents of these alkaloids in *Coptis* rhizome, which suggest that the poor oral absorption and bioavailability, and fast elimination rate of coptisine [38, 39].

Recently, re-purposing of the existing drugs for use in different disease attract the researcher because of cost-effectiveness [55–57]. Thus, it is noteworthy that the activity of *Coptis* rhizome in this study could be a promising re-purposing of Kampo medicine to formulate the treatment of malaria.

## Conclusions

In summary, this is the first study demonstrating the in vivo anti-malarial activity of *Coptis* rhizome and coptisine chloride. This finding suggests that *Coptis* rhizome is a potential natural resource for anti-malarial, promising drug re-purposing for malaria, and its active compound coptisine chloride could be a potential anti-malarial lead candidate.

## Supplementary information

**Additional file 1: Table S1. **List of crud drug extracts in Kampo library. **Table S2.** List of compounds in Kampo library. **Table S3.** In vitro anti-malarial activities against P.* falciparum* CQ/mefloquine (MQ)-sensitive (3D7) strains and the cytotoxicities using adult mouse brain cell (AMB) of crude drug extracts. **Table S4.** Formulation of herbal extracts percentage by weight in Orengedokuto (Toyama verses Tsumura composition of Kampo formula Orengedokuto). **Table S5.** The average percentage parasitaemia and suppression profile of Orengedokuto and Coptis rhizome. **Table S6.** Detection of Coptis rhizome and its bioactive compounds in mice fed with Orengedokuto and Coptis rhizome. **Figure S1.** LCMS chromatogram of Coptis rhizome and Orengedokuto treated mice plasma. **Table S7.** The average percentage parasitaemia and suppression profile of coptisine chloride.

## Data Availability

The datasets used and/or analysed during the current study are available from the corresponding author on reasonable request.

## Abbreviations

ACT: Artemisinin-base combination therapy
AMB: Adult Mouse Brain
CC_50_: 50% cytotoxic concentration
CDL: Curved desolvation line
CM: Complete media
CQ: Chloroquine
DW: Distilled water
ESI: Electrospray ionization
IC_50_: 50% inhibitory concentration
IP: Intraperitoneal
iRBC: Infected RBC
IT-TOF: Ion trap time-of-flight
LC–MS: Liquid chromatography–mass spectrometry
RFU: Relative fluorescence unit
SI: Selectivity index
UPW: Ultra-pure water
WHO: World Health Organization

## References

[1] WHO (2019). World malaria report 2019.

[2] Wilson KL, Flanagan KL, Prakash MD, Plebanski M (2019). Malaria vaccines in the eradication era: current status and future perspectives. Expert Rev Vaccines..

[3] Zucca M, Scutera S, Savoia D (2013). New chemotherapeutic strategies against malaria, leishmaniasis and trypanosomiases. Curr Med Chem.

[4] Fidock DA, Rosenthal PJ, Croft SL, Brun R, Nwaka S (2004). Antimalarial drug discovery: efficacy models for compound screening. Nat Rev Drug Discov..

[5] Mnzava AP, Macdonald MB, Knox TB, Temu EA, Shiff CJ (2014). Malaria vector control at a crossroads: public health entomology and the drive to elimination. Trans R Soc Trop Med Hyg.

[6] Foley M, Tilley L (1997). Quinoline antimalarials: mechanisms of action and resistance. Int J Parasitol.

[7] Achan J, Talisuna AO, Erhart A, Yeka A, Tibenderana JK, Baliraine FN (2011). Quinine, an old anti-malarial drug in a modern world: role in the treatment of malaria. Malar J..

[8] Monjol BE, Useh MF (2017). Detection of *Plasmodium falciparum* chloroquine resistance transporter (PfCRT) mutant gene amongst malaria-infected pregnant women in Calabar, Nigeria. Ann Parasitol..

[9] Payne D (1987). Spread of chloroquine resistance in *Plasmodium falciparum*. Parasitol Today..

[10] Antony HA, Parija SC (2016). Antimalarial drug resistance: an overview. Trop Parasitol..

[11] Sowunmi A, Akano K, Ntadom G, Ayede AI, Ibironke FO, Aderoyeje T (2017). Therapeutic efficacy and effects of artemisinin-based combination treatments on uncomplicated *Plasmodium falciparum* malaria-associated anaemia in Nigerian children during seven years of adoption as first-line treatments. Infect Dis Poverity..

[12] WHO. Antimalarial drug resistance in the Greater Mekong Subregion: how concerned should we be? Geneva: World Health Organization; 2017. http://www.who.int/malaria/media/drug-resistance-greater-mekong-qa/en/. Accessed 6 Mar 2018.

[13] Amato R, Pearson RD, Almagro-Garcia J, Amaratunga C, Lim P, Suon S (2018). Origins of the current outbreak of multidrug-resistant malaria in southeast Asia: a retrospective genetic study. Lancet Infect Dis..

[14] Imwong M, Suwannasin K, Kunasol C, Sutawong K, Mayxay M, Rekol H (2017). The spread of artemisinin-resistant *Plasmodium falciparum* in the Greater Mekong subregion: a molecular epidemiology observational study. Lancet Infect Dis..

[15] Li F-S, Weng J-K (2017). Demystifying traditional herbal medicine with modern approach. Nat Plants..

[16] Norn S, Permin H, Kruse PR, Kruse E (2009). From willow bark to acetylsalicylic acid. Dan Medicinhist Arbog..

[17] Whayne TF (2018). Clinical use of digitalis: a state of the art review. Am J Cardiovasc Drug..

[18] Norn S, Kruse PR, Kruse E (2005). History of opium poppy and morphine. Dan Medicinhist Arbog..

[19] Yuan H, Ma Q, Ye L, Piao G (2016). The traditional medicine and modern medicine from natural products. Molecules.

[20] Tajuddeen N, Van Heerden FR (2019). Antiplasmodial natural products: an update. Malar J..

[21] Permin H, Norn S, Kruse E, Kruse PR (2016). On the history of *Cinchona* bark in the treatment of malaria. Dan Medicinhistorisk Arbog..

[22] Shanks GD (2016). Problematic malaria prophylaxis with quinine. Am J Trop Med Hyg.

[23] Gachelin G, Garner P, Ferroni E, Tröhler U, Chalmers I (2017). Evaluating *Cinchona* bark and quinine for treating and preventing malaria. J Roy Soc Med..

[24] de Ridder S, van der Kooy F, Verpoorte R (2008). *Artemisia annua* as a self-reliant treatment for malaria in developing countries. J Ethnopharmacol.

[25] WHO. Traditional medicine. Geneva: World Health Organization; 2019. https://www.afro.who.int/health-topics/traditional-medicine. Accessed 1 Apr 2019.

[26] Pan S-Y, Litscher G, Gao S-H, Zhou S-F, Yu Z-L, Chen H-Q (2014). Historical perspective of traditional indigenous medical practices: the current renaissance and conservation of herbal resources. Evid Based Complementary Altern Med..

[27] Hoffmann KM, Herbrechter R, Ziemba PM, Lepke P, Beltrán L, Hatt H (2016). Kampo medicine: evaluation of the pharmacological activity of 121 herbal drugs on GABAA and 5-HT3A receptors. Front Pharmacol..

[28] Chen X, Xiang L, Shi L, Li G, Yao H, Han J (2017). Identification of crude drugs in the Japanese pharmacopoeia using a DNA barcoding system. Sci Rep..

[29] Arai M, Katai S, Muramatsu S-I, Namiki T, Hanawa T, Izumi S-I (2012). Current status of Kampo medicine curricula in all Japanese medical schools. BMC Complem Altern Med..

[30] Yakubo S, Ito M, Ueda Y, Okamoto H, Kimura Y, Amano Y (2014). Pattern classification in Kampo medicine. Evid Based Complemen Altern Med..

[31] Yoshino T, Katayama K, Horiba Y, Munakata K, Yamaguchi R, Imoto S (2016). Predicting Japanese Kampo formulas by analyzing database of medical records: a preliminary observational study. BMC Med Inform Decis Mak.

[32] Motoo Y, Seki T, Tsutani K (2011). Traditional Japanese medicine, Kampo: its history and current status. Chin J Integr Med..

[33] Watanabe K, Matsuura K, Gao P, Hottenbacher L, Tokunaga H, Nishimura K (2011). Traditional Japanese Kampo medicine: clinical research between modernity and traditional medicine—the state of research and methodological suggestions for the future. Evid Based Complementary Altern Med..

[34] Trager W, Jensen JB (1978). Cultivation of malarial parasites. Nature.

[35] Borenfreund E, Babich H (1987). In vitro cytotoxicity of heavy metals, acrylamide, and organotin salts to neural cells and fibroblasts. Cell Biol Toxicol.

[36] TradMPD. Traditional Medical & Pharmaceutical Database. http://dentomed.toyama-wakan.net/en/information_on_experimental_crude_drug_extracts/. Accessed 1 May 2020.

[37] Mosaddeque F, Mizukami S, Kamel MG, Teklemichael AA, Dat TV, Mizuta S (2018). Prediction model for antimalarial activities of hemozoin inhibitors by using physicochemical properties. Antimicrob Agents Chemother.

[38] Yan Y, Zhang H, Zhang Z, Song J, Chen Y, Wang X (2017). Pharmacokinetics and tissue distribution of coptisine in rats after oral administration by liquid chromatography-mass spectrometry. Biomed Chromatogr..

[39] Su J, Miao Q, Miao P, Zhao Y, Zhang Y, Chen N (2015). Pharmacokinetics and brain distribution and metabolite identification of coptisine, a protoberberine alkaloid with therapeutic potential for CNS disorders, in rats. Biol Pharm Bull.

[40] Parhizgar AR, Tahghighi A (2017). Introducing new antimalarial analogues of chloroquine and amodiaquine: a narrative review. Iran J Med Sci..

[41] Cañón M, Diaz H, Olarte A (2017). Mathematical model for the spread of drug resistance in *Plasmodium falciparum* parasite considering transmission conditions. J Theor Biol.

[42] Nsanzabana C, Djalle D, Guérin PJ, Ménard D, González IJ (2018). Tools for surveillance of anti-malarial drug resistance: an assessment of the current landscape. Malar J..

[43] Fujii A, Okuyama T, Wakame K, Okumura T, Ikeya Y, Nishizawa M (2017). Identification of anti-inflammatory constituents in *Phellodendri* cortex and *Coptidis* Rhizoma by monitoring the suppression of nitric oxide production. J Nat Med.

[44] Oshima N, Shimizu T, Narukawa Y, Hada N, Kiuchi F (2018). Quantitative analysis of the anti-inflammatory activity of orengedokuto II: berberine is responsible for the inhibition of NO production. J Nat Med.

[45] Chen Y, Xian Y, Lai Z, Loo S, Chan WY, Lin Z-X (2016). Anti-inflammatory and anti-allergic effects and underlying mechanisms of Huang-Lian-Jie-Du extract: implication for atopic dermatitis treatment. J Ethnopharmacol.

[46] Nonaka M, Murata Y, Takano R, Han Y, Kabir MHB, Kato K (2018). Screening of a library of traditional Chinese medicines to identify anti-malarial compounds and extracts. Malar J..

[47] Wilson DW, Langer C, Goodman CD, McFadden GI, Beeson JG (2013). Defining the timing of action of antimalarial drugs against *Plasmodium falciparum*. Antimicrob Agents Chemother.

[48] Mahmoudvand H, Ayatollahi Mousavi SA, Sepahvand A, Sharififar F, Ezatpour B (2014). Antifungal, antileishmanial, and cytotoxicity activities of various extracts of *Berberis vulgaris* (Berberidaceae) and its active principle Berberine. ISRN Pharmacol..

[49] Chou H-C, Lu Y-C, Cheng C-S, Chen Y-W, Lyu P-C, Lin C-W (2012). Proteomic and redox-proteomic analysis of berberine-induced cytotoxicity in breast cancer cells. J Proteomics..

[50] El Khalki L, Tilaoui M, Jaafari A, Ait Mouse H, Zyad A (2018). Studies on the dual cytotoxicity and antioxidant properties of *Berberis vulgaris* extracts and its main constituent berberine. Adv Pharmacol Sci.

[51] Han B, Jiang P, Li Z, Yu Y, Huang T, Ye X (2018). Coptisine-induced apoptosis in human colon cancer cells (HCT-116) is mediated by PI3K/Akt and mitochondrial-associated apoptotic pathway. Phytomedicine.

[52] Fu S, Ni S, Wang D, Hong T (2018). Coptisine suppresses mast cell degranulation and ovalbumin-induced allergic rhinitis. Molecules.

[53] Shi L-L, Jia W-H, Zhang L, Xu C-Y, Chen X, Yin L (2019). Glucose consumption assay discovers coptisine with beneficial effect on diabetic mice. Eur J Pharmacol.

[54] Xu Z, Feng W, Shen Q, Yu N, Yu K, Wang S, Chen Z (2017). Rhizoma coptidis and berberine as a natural drug to combat aging and aging-related diseases via anti-oxidation and AMPK activation. Aging Dis..

[55] Pushpakom S, Iorio F, Eyers PA, Escott KJ, Hopper S, Wells A (2019). Drug repurposing: progress, challenges and recommendations. Nat Rev Drug Discov..

[56] Corsello SM, Bittker JA, Liu Z, Gould J, McCarren P, Hirschman JE (2017). The Drug Repurposing Hub: a next-generation drug library and information resource. Nat Med.

[57] Rabinovich NR (2018). Ivermectin: repurposing an old drug to complement malaria vector control. Lancet Infect Dis..

