# Supplementary material for: Anti-malarial activity of traditional Kampo medicine Coptis rhizome extract and its major active compounds
Source: Malar J. 2020 Jun 8;19:204. doi: 10.1186/s12936-020-03273-x (PMC7282140; doi:10.1186/s12936-020-03273-x)
Supplement: Supplementary file 1 — Additional file 1: Table S1. List of crud drug extracts in Kampo library. Table S2. List of compounds in Kampo library. Table S3. In vitro anti-malarial activities against P. falciparum CQ/mefloquine (MQ)-sensitive (3D7) strains and the cytotoxicities using adult mouse brain cell (AMB) of crude drug extracts. Table S4. Formulation of herbal extracts percentage by weight in Orengedokuto (Toyama verses Tsumura composition of Kampo formula Orengedokuto). Table S5. The average percentage parasitaemia and suppression profile of Orengedokuto and Coptis rhizome. Table S6. Detection of Coptis rhizome and its bioactive compounds in mice fed with Orengedokuto and Coptis rhizome. Figure S1. LCMS chromatogram of Coptis rhizome and Orengedokuto treated mice plasma. Table S7. The average percentage parasitaemia and suppression profile of coptisine chloride. [file 12936_2020_3273_MOESM1_ESM.docx]

**Additional files**

**Additional file: Table S1****. List of crud drug extracts in Kampo library.**

| ID.No. | Crude drug extract name | ID.No. | Crude drug extract name | ID.No. | Crude drug extract name |
| --- | --- | --- | --- | --- | --- |
| 1 | *Clematis root* | 41 | *Asiasarum root* | 81 | *Citrus unshiu peel* |
| 2 | *Artemisia capillaris flower* | 42 | *Saffron* | 82 | *Gastrodia tuber* |
| 3 | *Turmeric* | 43 | *Smilax rhizome* | 83 | *Asparagus root* |
| 4 | *Lindera root* | 44 | *Gardenia fruit* | 84 | *Benincasa seed* |
| 5 | *Corydalis tuber* | 45 | *Panax notoginseng Root* | 85 | *Angelica dahurica root* |
| 6 | *Astragalus root* | 46 | *Cornus fruit* | 86 | *Codonopsis root* |
| 7 | *Scutellaria root* | 47 | *Japanese zanthoxylum peel* | 87 | *Peach kernel* |
| 8 | *Phellodendron bark* | 48 | *Jujube seed* | 88 | *Aralia rhizome* |
| 9 | *Cherry bark* | 49 | *Dioscorea rhizome* | 89 | *Eucommia bark* |
| 10 | *Coptis rhizome* | 50 | *Sparganium rhizome* | 90 | *Cistanche herb* |
| 11 | *Polygala root* | 51 | *Rehmannia root* | 91 | *Ginseng* |
| 12 | *Artemisiae leaf* | 52 | *Eleutherococcus senticosus rhizome* | 92 | *Fritillaria bulb* |
| 13 | *Polygonum root* | 53 | *Lycium Bark* | 93 | *Densefruit Pittany Root Bark* |
| 14 | *Curcuma Rhizome* | 54 | *Tribulus fruit* | 94 | *Ophiopogon root* |
| 15 | *Pueraria root* | 55 | *Peony root* | 95 | *Mentha herb* |
| 16 | *Trichosanthes root* | 56 | *Plantago seed* | 96 | *Pinellia tuber* |
| 17 | *Processed ginger* | 57 | *Processed rehmannia root* | 97 | *Japanese angelica root* |
| 18 | *Glycyrrhiza* | 58 | *Amomum seed* | 98 | *Atractylodes rhizome* |
| 19 | *Platycodon root* | 59 | *Ginger* | 99 | *Loquat leaf* |
| 20 | *Chrysanthemum flower* | 60 | *Cimicifuga rhizome* | 100 | *Areca* |
| 21 | *Immature orange* | 61 | *Magnolia flower* | 101 | *Poria sclerotium* |
| 22 | *Notopterygium* | 62 | *Gentiana macrophylla root* | 102 | *Sinomenium stem and rhizome* |
| 23 | *Apricot kernel* | 63 | *Red peony root* | 103 | *Imperata rhizome* |
| 24 | *Sophora root* | 64 | *Cnidium rhizome* | 104 | *Saposhnikovia root and rhizome* |
| 25 | *Sasa leaf* | 65 | *Ginseng* | 105 | *Processed aconite root* |
| 26 | *Schizonepeta spike* | 66 | *Atractylodes lancea rhizome* | 106 | *Quercus bark* |
| 27 | *Suberect spatholobus tem* | 67 | *Mulberry bark* | 107 | *Malaytea scurfpea fruit* |
| 28 | *Cinnamom Bark* | 68 | *Mulberry Leaf* | 108 | *Moutan Bark* |
| 29 | *Scrophularia root* | 69 | *Dipsacus root* | 109 | *Ephedra herb* |
| 30 | *Safflower* | 70 | *Perilla herb* | 110 | *Hemp fruit* |
| 31 | *Silktree albizia bark* | 71 | *Rhubarb* | 111 | *Shrub chaste tree fruit* |
| 32 | *Red Ginseng* | 72 | *Jujube* | 112 | *Saussurea root* |
| 33 | *Cyperus rhizome* | 73 | *Alisma tuber* | 113 | *Myrrh* |
| 34 | *Magnolia bark* | 74 | *Salvia miltiorrhiza root* | 114 | *Bitter cardamon* |
| 35 | *Achyranthes root* | 75 | *Bamboo culm* | 115 | *Leonurus herb* |
| 36 | *Euodia fruit* | 76 | *Panax japonicus rhizome* | 116 | *Coix seed* |
| 37 | *Burdock fruit* | 77 | *Anemarrhena rhizome* | 117 | *Japanese gentian* |
| 38 | *Sesame* | 78 | *Clove* | 118 | *Alpinia officinarum rhizome* |
| 39 | *Schisandra fruit* | 79 | *Uncaria hook* | 119 | *Ganoderma* |
| 40 | *Bupleurum root* | 80 | *Polyporus sclerotium* | 120 | *Forsythia rruit* |

**Additional file: Table S2. List of compounds in Kampo library.**

| **ID.No.** | **Compound name** | **ID.No.** | **Compound name** | **ID.No.** | **Compound name** |
| --- | --- | --- | --- | --- | --- |
| **1** | Acontine | 33 | Dehydrocorydaline nitrate | 65 | (*Z*)-Ligustilide |
| **2** | Albiflorin | 34 | Dehydrocostuslactone | 66 | Limonin |
| **3** | Alisol A | 35 | Demethoxycurcumine | 67 | Liquiritin |
| **4** | Alison B | 36 | Dihydrocapsaicin | 68 | Loganin |
| **5** | Alkanin | 37 | *Dimethylsculetin* | 69 | Luteolin |
| **6** | Amygdalin | 38 | Eleutheroside B | 70 | Magnolol |
| **7** | Arbutin | 39 | (-)-Epigallocathechin gallate | 71 | Mesaconitine |
| **8** | Astragaloside IV | 40 | Epihesperidine | 72 | Naringin |
| **9** | Atractylenolide III | 41 | Ergosterol | 73 | Nodakenin |
| **10** | Atractylodin | 42 | β- Eudesmol | 74 | Osthol |
| **11** | Atropin sulphate | 43 | Evodiamine | 75 | Oxymatrine |
| **12** | Aucubin | 44 | (*E*)-Ferulic acid | 76 | Paeoniflorin |
| **13** | Baicalein | 45 | Geniposide | 76 | Paeoniflorin |
| **14** | Baicalin | 46 | Geniposidic acid | 77 | Paeonol |
| **15** | Barbaloin | 47 | Gentiopicroside | 78 | Palmatine chloride |
| **16** | Benzoylmesaconine hydrochloride | 48 | (6)-Gingerol | 79 | Perillaldhyde |
| **17** | Berberin chloride | 49 | Ginsenoside-Rb1 | 81 | Puerarin |
| **18** | Bergenin | 50 | Ginsenoside-Rc | 82 | Rhynchophylline |
| **19** | Bisdemethoxycurcumin | 51 | Ginsenoside-Rd | 83 | Rosmaric Acid |
| **20** | Bufalin | 52 | Ginsenoside-Re | 84 | Saikosaponin a |
| **21** | Bufotalin | 53 | Ginsenoside-Rg1 | 85 | Saikosaponin b2 |
| **22** | Cappillarisin | 54 | Glabridine | 86 | Saikosaponin c |
| **23** | (*E*)-Capsaicin | 55 | Glycyrrhizic acid | 87 | Saikosaponin d |
| **24** | Catalpol | 56 | Gomison A | 88 | Schizandrin |
| **25** | (*E*)-Chlorogenic acid | 57 | Gomison N | 89 | Sennoside A |
| **26** | (*E*)-Cinnamic acid | 58 | Hesperidin | 90 | Sennoside B |
| **27** | Cinobufagin | 59 | Hirsutine | 91 | Shikonin |
| **28** | Cinobufotalin | 60 | Honokiol | 92 | (6)-Shogaol |
| **29** | Coptisine chloride | 61 | Hypacontine | 93 | Sinomenine |
| **30** | Corydalin | 62 | Icariin | 94 | Swertiamarin |
| **31** | Costunolide | 63 | Isofraxidine | 95 | Timosaponin A-III |
| **32** | Curcumin | 64 | Isorhynchophyllin | 96 | Wogonin |

**Additional file: Table S3. *In vitro* antimalarial activities against *P. falciparum CQ/mefloquine (MQ)-sensitive (3D7) strains* and the cytotoxicities using adult mouse brain (AMB) cell of crude drug extracts.**

| Name | IC_50_ (µg/mL) | CC_50_ (µg/mL) | SI |
| --- | --- | --- | --- |
| *Coptis rhizome* | 1.9 | > 500 | >263 |
| *Phellodendron bark* | 6.7 | 23.5 | 3.5 |
| *Clove* | 18.5 | > 500 | >27 |
| *Quercus bark* | 44 | > 500 | >11.7 |
| *Rhubarb* | 61.3 | > 500 | >8.2 |
| *Sesame* | 81.2 | > 500 | >6.1 |

IC_50_: 50% inhibitory concentration.

CC_50_: 50% cytotoxic concentration using adult mouse brain cells

SI: Selectivity Index

Values are the mean from two independent experiments performed in duplicate.

**Additional file: Table S4. Formulation of herbal extracts percentage by weight in Orengedokuto (Toyama verses Tsumura composition of Kampo formula Orengedokuto).**

| Formula (Company) | Herbal extracts | % |
| --- | --- | --- |
| Orengedokuto (Toyama) | *Coptise rhizome* | 33.3 |
|  | *Phellodendron bark* | 22.2 |
|  | *Scutellaria root* | 22.2 |
|  | *Gardenia fruit* | 22.2 |
| Orengedokuto (Tsumura) | *Coptise rhizome* | 23.5 |
|  | *Phellodendron bark* | 17.6 |
|  | *Scutellaria root* | 35.2 |
|  | *Gardenia fruit* | 23.5 |

The content of crude drug in a formulated Orengedokuto calculated based on their weight in gram. The information obtained from University of Toyama, traditional medicine, and pharmaceutical data base. Source: <http://dentomed.toyama-wakan.net/en/information_on_experimental_crude_drug_extracts/> and Tsumura company: https://www.tsumura.co.jp/products/qr_lp/english.html.

**Additional file: Table S5. The average percentage parasitemia and suppression profile of Orengedokuto and *Coptis rhizome*.**

| Treatment | Dose in mg/kg/day | % parasitemia  (%suppression) | | | | |
| --- | --- | --- | --- | --- | --- | --- |
|  |  | Day 3 | Day 4 | Day 5 | Day 6 | Day 7 |
| Orengedokuto | 365 | 0.60 ± 0.20  (23.18) | 2.89 ± 0.63  (42.48) | 12.07 ± 0.42  (22.13) | 16.87 ± 2.65  (11.25) | 13.75 ± 4.91  (8.07) |
| *Coptis rhizome* | 122 | **0.34 ± 0.16  (59.25) | *2.48 ± 0.82  (50.38) | **6.96 ± 0.39  (55.21) | **5.66 ± 1.35  (70.57) | **3.94 ± 0.87  (72.13) |
| NC (DW) | 0.2 mL | 0.85 ± 0.46  (0) | 5.11 ± 0.52  (0) | 15.54 ± 0.84  (0) | 19.32 ± 5.23  (0) | 14.72 ± 2.80  (0) |
| PC (CQ) | 10 | 0.1 ± 0.022  (100) | 0.0005 + 0.001  (100) | 0  (100) | 0  (100) | 0  (100) |

Data are presented mean + SD (five mice for each group). The average percentage parasitemia and suppression activity of CR and OGT treated C57BL/6N mice infected with a mefloquine-resistant and CQ-sensitive strain of *P. yoelii*. CR: *Coptis rhizome*, OGT: Orengedokuto, NC (DW): (Negative control, Distilled water), PC (CQ): (Positive control, Chloroquine).

* P value < 0.05, ** P value < 0.01

**Additional file: Table S6. Detection of *Coptis rhizome* and its bioactive compounds in mice fed with Orengedokuto and *Coptis rhizome*.**

| **Compounds** | ***m/z*** | ***Coptis rhizome* ext.** | **Plasma from**  **Orengedokuto fed mice** | | | | | **Plasma from**  ***Coptis rhizome* fed mice** | | | | |
| --- | --- | --- | --- | --- | --- | --- | --- | --- | --- | --- | --- | --- |
|  |  |  | 1 | 2 | 3 | 4 | 5 | 1 | 2 | 3 | 4 | 5 |
| **Berberine** | 336.1 | ○ | ○ | ○ | ○ | ○ | ○ | ○ | ○ | ○ | - | ○ |
| **Palmatine** | 352.1 | ○ | ○ | ○ | ○ | - | - | ○ | ○ | - | - | ○ |
| **Coptisine** | 320.1 | ○ | - | - | ○ | - | - | ○ | ○ | - | - | ○ |

Detection of *Coptise rhizome* and its bioactive compounds namely berberine, palmatine and coptisine after the mice received oral administration of *Coptis rhizome* and Orengedokuto with a doses of 122mg/kg and 365mg/kg for each group, respectively. Five mice were used for each groups. The blood was collected 1hr after administration and plasma concentration was measured by LC-MS.

〇: the signal of compound was detected. 　 **-**: the signal was not detected.

**Additional file: Figure S1. LCMS chromatogram of *Coptis rhizome* and Orengedokuto treated mice plasma.**

1. *Coptis rhizome*

TIC

EIC *m/z* 336.1

EIC *m/z* 352.1

EIC *m/z* 320.1

1. Orengedokuto

TIC

EIC *m/z* 336.1

EIC *m/z* 352.1

EIC *m/z* 320.1

LCMS chromatogram of *Coptis rhizome* and Orengedokuto treated mice plasma. The plasma samples from *Coptis rhizome* (a, 122 mg/kg) and Orengedokuto (b, 365 mg/kg) administrated mice were analysed. Blood samples were collected after 1 hr of oral administration. For each chromatogram, top shows total ion chromatogram (TIC). Second to forth chromatogram show the extracted ion chromatogram (EIC) at *m/z* 336, 352, and 320, respectively. The signal intensities for three EIC are magnified 10 times of TIC. The arrows indicate the signals of berberine (*m/z* 336.1, *t*_R_ 14.0 min.), palmatine (*m/z* 352.1, *t*_R_ 13.8 min.), and coptisine (*m/z* 320.1, *t*_R_ 13.1 min.), respectively.

**Additional file: Table S7. The average percentage parasitemia and suppression profile of coptisine chloride**

| Treatment | Dose in mg/kg/day | % parasitemia  (% suppression) | | | |
| --- | --- | --- | --- | --- | --- |
|  |  | Day 3 | Day 4 | Day 5 | Day 6 |
| Coptisine chloride | 30 | **0.075 ± 0.04  (89) | **0.45 ± 0.24  (87) | ***1.70 ± 0.43  (82) | ***2.6 ± 0.89  (81) |
| NC (DW) | 0.2 mL | 0.69 ± 0.16  (0) | 3.42 ± 0.75  (0) | 9.2 ± 1.48  (0) | 14.0 ± 0.79  (0) |
| PC (CQ) | 10 | 0  (100) | 0  (100) | 0  (100) | 0  (100) |

Data are presented mean + SD (five mice for each group). The average percentage parasitemia and suppression activity of CC treated C57BL/6N mice infected with a mefloquine-resistant and CQ-sensitive strain of *P. yoelii*. CC: Coptisine chloride, NC (DW): (Negative control, distilled water), PC (CQ): (Positive control, Chloroquine).

* P value < 0.05, ** P value < 0.01, ***p value < 0.001
